# Activation of the cAMP Pathway Induces RACK1-Dependent Binding of β-Actin to *BDNF* Promoter

**DOI:** 10.1371/journal.pone.0160948

**Published:** 2016-08-09

**Authors:** Jeremie Neasta, Anna Fiorenza, Dao-Yao He, Khanhky Phamluong, Patrick A. Kiely, Dorit Ron

**Affiliations:** 1 Department of Neurology, University of California, San Francisco, California, United States of America; 2 Graduate Entry Medical School, Health Research Institute and Materials and Surface Science Institute, University of Limerick, Limerick, Ireland; University of Louisville, UNITED STATES

## Abstract

RACK1 is a scaffolding protein that contributes to the specificity and propagation of several signaling cascades including the cAMP pathway. As such, RACK1 participates in numerous cellular functions ranging from cell migration and morphology to gene transcription. To obtain further insights on the mechanisms whereby RACK1 regulates cAMP-dependent processes, we set out to identify new binding partners of RACK1 during activation of the cAMP signaling using a proteomics strategy. We identified β-actin as a direct RACK1 binding partner and found that the association between β-actin and RACK1 is increased in response to the activation of the cAMP pathway. Furthermore, we show that cAMP-dependent increase in *BDNF* expression requires filamentous actin. We further report that β-actin associates with the *BDNF* promoter IV upon the activation of the cAMP pathway and present data to suggest that the association of β-actin with *BDNF* promoter IV is RACK1-dependent. Taken together, our data suggest that β-actin is a new RACK1 binding partner and that the RACK1 and β-actin association participate in the cAMP-dependent regulation of *BDNF* transcription.

## Introduction

RACK1 is a ubiquitous scaffolding protein that belongs to the tryptophan-aspartate (WD-40) repeat family of proteins [1, 2]. A critical feature of RACK1 is its multifaceted and compact propeller structure, which allows the protein to associate with several binding partners at the same time [1, 2], and this characteristic makes RACK1 a unique molecular node with the capacity for integrating and converting several signals into a cellular response.

RACK1 was originally identified as a βII protein kinase C (βII PKC) binding protein that targets the active kinase to its proper subcellular location [3]. Through its association with numerous other signaling molecules, RACK1 was later found to participate in the propagation and accuracy of other major cascades including the cAMP signaling pathway [1, 2]. For example, RACK1 binds the cAMP-specific degrading hydrolase, phosphodiesterase 4D5 (PDE4D5) [4], and provides a focal point for the cross-talk between the PKC and cAMP signaling cascades [5]. In addition, RACK1 facilitates the transcription of genes via a cAMP-dependent translocation of the scaffolding protein to the nucleus [6–9]. Specifically, activation of cAMP signaling leads to the sequestration of RACK1 in the nucleus where the scaffolding protein associates with the *brain-derived neurotrophic factor* (*BDNF*) promoter IV, resulting in epigenetic-dependent increase in the transcription of the neurotrophic factor [6].

As RACK1 signals by means of protein-protein association, deciphering its interaction network in response to an activation of specific signaling cascades is a critical step towards understanding the cellular processes that are regulated by the scaffolding protein.

Here, we set out to identify cAMP-responsive binding partners of RACK1. We found that RACK1 directly interacts with β-actin and that the association between the two proteins is increased upon activation of cAMP signaling. Importantly, our data also suggest that a functional interplay between RACK1 and nuclear β-actin regulates cAMP-induced *BDNF* transcription.

## Material and Methods

### Materials

Anti-RACK1 (sc-17754), anti-pan actin (sc-1616), anti-GAPDH (sc-25778) antibodies and the horseradish peroxidase (HRP)-conjugated secondary antibodies were purchased from Santa Cruz Biotechnology. Anti-phospho-CREB (#9191), and anti-CREB (#9121) antibodies were from Cell Signaling. Anti-β-actin antibody (#A5316, clone AC-74), forskolin, (FSK) cytochalasin D and phosphatase inhibitor cocktails 1 and 2 were purchased from Sigma-Aldrich. The protease inhibitor mixture was purchased from Roche Applied Science. Nuclear proteins were isolated using the NE-PER Nuclear and Cytoplasmic Extraction Reagents from Thermo Scientific. Trypsin, and the reverse transcription system and 2X PCR Master Mix were purchased from Promega and SYBR® Green PCR Master Mix was from Applied Biosystem. Primers for PCR were synthesized by Sigma-Genosys. Chromatin immunoprecipitation (ChIP) assay kit was from Millipore. Amylose resin was from New England BioLabs. Deep Purple™ Total Protein Stain and Enhanced Chemiluminescence (ECL) were from GE Healthcare. Non-muscle purified actin >99% purity (#APHL99) was purchased from Cytoskeleton. NuPAGE® Bis-Tris pre-casted gels and recombinant protein G-agarose were from Invitrogen.

### Cell culture

SH-SY5Y human neuroblastoma cells were cultured in Dulbecco’s modified Eagle medium (DMEM) containing 10% fetal bovine serum (FBS) supplemented with non-essential amino acid solution. Cells were incubated in a low-serum medium containing 1% FBS for at least 24 h before treatment.

### RACK1 immunoprecipitation and gel staining

For RACK1 immunoprecipitation from total cell extract, SH-SY5Y cells were lysed in immunoprecipitation (IP) buffer (1% Triton X-100, 150 mM NaCl, 10 mM Tris HCl, pH 7.4, 1 mM EDTA, 1 mM EGTA, and protease and phosphatase inhibitor cocktails).

For RACK1 immunoprecipitation from nuclear extract, SH-SY5Y were rinsed twice with ice-cold phosphate buffered saline and spun down for 1 min at 500 g. Nuclear proteins were isolated using the NE-PER Nuclear and Cytoplasmic Extraction Reagents according to the provider’s instructions, then diluted in IP buffer.

Nuclear or total cell extract diluted in IP buffer was pre-cleared and incubated with anti-RACK1 antibody or IgG. After overnight incubation at 4°C, recombinant protein G-agarose was added, and the mixture was further incubated at 4°C for 2 h. The agarose resin was extensively washed with IP buffer, the immunoprecipitated proteins were eluted in loading buffer (2% SDS, 23% β-mercaptoethanol, 50 mM Tris HCl, pH 6.8, 10% glycerol, 1 mm EDTA, 0.1% bromophenol blue), and resolved by SDS-PAGE. Gels were stained with Deep Purple™ Total Protein Stain according to the provider’s instructions and scanned with Typhoon™ scanner (GE Healthcare) (excitation 532 nm, emission 610BP filter).

### Mass spectrometry identification of proteins

Identification of proteins for RACK1 IP from total cell extract was conducted as previously described [9]. Briefly, whole lanes of interest (IgG versus RACK1) were manually excised and cut into small pieces from a representative gel. Identification of proteins for RACK1 IP from nuclear extract was conducted on the gel slice of interest. Proteins were subjected to trypsin digestion and mixtures of proteolytic peptides were on-line separated by nanoLC utilizing the Eksigent 2D LC NanoLC System (Eksigent/AB Sciex) interfaced with the QStar XL mass spectrometer (Applied Biosystems Sciex). ProteinPilot™ Software 4.0, which utilizes the Paragon™ Algorithm (Applied Biosystems Sciex), was used for peak detection, mass peak list generation and database searches. Protein identifications based on multiple peptides were accepted using a cut-off score of 1.69897 that represented more than 98% confidence.

### Western blot analysis

Proteins were resolved on a 4%-12% SDS-PAGE gel and were transferred onto nitrocellulose membranes. Membranes were blocked for 1 h with 5% (*w/v*) nonfat milk in Tris Buffered Saline containing 0.1% (*v/v*) Tween 20 (TBS-T), then incubated overnight at 4°C in the blocking solution including the appropriate antibody. After extensive washes with TBS-T, bound primary antibodies were detected with HRP-conjugated secondary antibodies and visualized by ECL.

### MBP Column Assay

MBP and MBP-RACK1 constructs were previously described [10]. Recombinant proteins were expressed in *Escherichia coli* by the induction with 1.5 mM isopropyl β-D-1-thiogalactopyranoside (IPTG) for 4 h. Cells were then lysed by successive freeze/thaw cycles and sonication. Crude extracts of MBP-fusion proteins were incubated with 1 ml pre-washed amylose suspension, and the column was washed extensively with column buffer (20 mm Tris-HCl, pH 7.5, 200 mm NaCl, 1 mm EDTA, 0.002% sodium azide, and 10 mm β-mercaptoethanol). SH-SY5Y lysate (1 mg), diluted to 1 μg/μl in IP buffer, was applied to the column for 1 h at room temperature. After washes with 30 ml of a solution containing 50 mm Tris-HCl, pH 7.5, 0.1% polyethylene glycol (*Mr* 15000–20000), 1.2 mm β-mercaptoethanol and 0.2 m NaCl, bound proteins were eluted 3 times with 500 μl of column buffer supplemented with 50 mm maltose and resolved by SDS-PAGE. Proteins were stained with colloidal Coomassie blue (G-250) or further analyzed by western blot.

For actin binding assay, 25 μg of >99% purified non-muscle actin (a mix of 85% and 15% of β-actin and γ-actin respectively according to the provider) diluted in 1 ml of IP buffer was applied to purified MBP or MBP-RACK1 immobilized on amylose resin. After 2 h incubation at room temperature, amylose resin was washed as described above. Bound proteins were eluted with loading buffer or 50 mm maltose and analyzed by western blot.

### Reverse transcription and semi-quantitative polymerase chain reaction (RT-PCR)

Total RNA was isolated using TRIzol reagent and 1 μg was reverse transcribed via the reverse transcription system, with incubation at 42°C for 30 min followed by 6 min at 95°C. Gene expression was analyzed by semi-quantitative PCR with temperature cycling parameters consisting of initial denaturation at 94°C for 2 min followed by cycles of denaturation at 94°C for 30 s, annealing at 58°C for 30 s, extension at 72°C for 2 min, and a final incubation at 72°C for 7 min. PCR products were resolved on a 1.8% agarose gel in Tris/acetic acid/EDTA buffer with 0.25 μg/ml ethidium bromide and photographed by Eagle Eye II (Stratagen, La Jolla, CA). The images were digitally scanned and the signals were quantified by densitometry using the NIH Image 1.63 program. The following primers were used for the PCR analysis: BDNF-Forward 5’-CTT TGG TTG CAT GAA GGC TGC-3’, BDNF-Reverse 5’-GTC TAT CCT TAT GAA TCG CCA G-3’, GAPDH-Forward 5’-TGA AGG TCG GTG TGA ACG GAT TTG GC-3’ and GAPDH-Reverse 5’- CAT GTA GGC CAT GAG GTC CAC CAC-3’.

Semi-quantitative PCR analysis of the chromatin immunoprecipitation (ChIP) samples was conducted using the following *BDNF* PIV primers: forward 5’-AAG CAT GCA ATG CCC TGG AAC-3’ and reverse 5’-TGC CTT GAC GTG CGC TGT CAT-3’. Products of ChIP-PCR were separated on a 2% agarose gel with ethidium bromide, and the PCR signals were quantified using NIH Image 1.63.

### Quantitative PCR (qPCR)

qPCR of ChIP samples was performed in an Applied Biosystems 7900 real-time PCR unit using SYBR® Green PCR Master Mix following manufacturer’s instructions. Each independent sample was assayed in triplicate. The primer sequences used in the ChIP-qPCR assays are: *BDNF* PIV: forward 5’-CCC TGG AAC GGA ACT CTT CTA AT-3’ and reverse 5’-CCG CTG CCT CGA AAT AGA C-3’.

### Chromatin immunoprecipitation (ChIP) assay

ChIP was conducted using the Millipore ChIP assay kit following the manufacturer’s protocol. Briefly, cells were cross-linked with 1% paraformaldehyde and lysed in 1% SDS containing buffer. After gentle sonication, protein concentration was determined by BCA assay and the same amount of cross-linked chromatin was incubated with 5 μg of anti-β-actin (clone AC-74) antibody, which was previously used for this application [11]. DNA-β-actin complex was immunoprecipitated with recombinant protein G-agarose, reverse cross-linked and DNA was extracted by ethanol precipitation. DNA from ChIP samples was then analyzed by semi-quantitative or quantitative PCR (qPCR).

### Cloning and Preparation of Recombinant Adenoviruses

The preparation of the shRACK1 recombinant adenoviruses was previously described [6]. The shRACK1 sequence GAC CAT CAT CAT GTG GAA GC used to knockdown RACK1 was previously validated in SH-SY5Y cells [6]. A recombinant adenovirus expressing the non-related 19-nt sequence (shCT), ATG AAC GTG AAT TGC TCA A, was used as control [6]. Viruses were amplified in HEK293 cells, then purified using the Adeno-X^TM^ Maxi Purification kit, and tittered with the Adeno-X Rapid Titer kit (Clontech). SH-SY5Y cells were infected with the virus 3 days before CHIP assay. Viruses were used at a concentration of 1 × 10^6^ infection units/ml.

### Data analysis

Depending of the design of the experiment, data were analyzed with one-way ANOVA or two-way ANOVA. Significant main effects and interactions of the ANOVAs were further investigated with the Newman-Keuls post hoc test or with two-tailed unpaired t-test. Statistical significance was set at p < 0.05. Data are presented as mean ± SEM.

## Results

### Mass spectrometry identification of actin as a RACK1 binding partner

**Fig 1:**
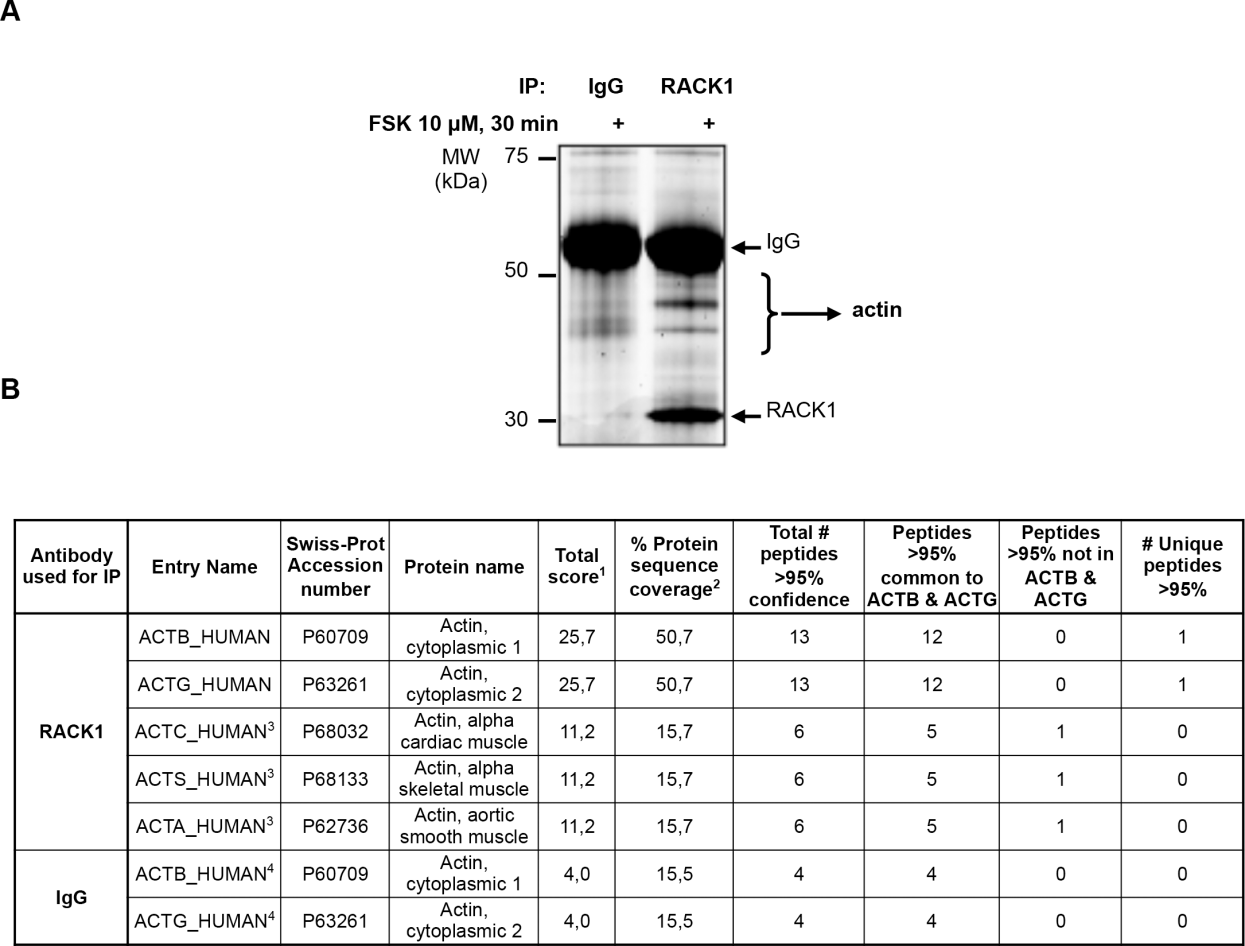
Mass spectrometry identification of actin as a binding partner of RACK1. ***A*** SH-SY5Y cells were treated with 10 μM FSK for 30 min, RACK1 was IPed from cell lysate and proteins were resolved by SDS-PAGE which was stained with Deep Purple™ to visualize proteins. Gel slices from both RACK1 and control IgG IPs were digested in-gel and the resulting peptides were submitted to tandem mass spectrometry (MS/MS) sequencing for protein identification, n = 2. ***B*** Identification of actin as a putative RACK1 binding protein. β-actin (ACTB), γ-actin (ACTG), alpha skeletal muscle actin (ACTS), aortic smooth muscle actin (ACTA), alpha cardiac muscle actin (ACTC) were identified in the RACK1 IP. A sum of score of peptides that were matched to the best hit within the family of homologous protein sequences (^**1**^). Calculation of the percent sequence coverage included all peptides matched to the protein, i.e., both unique and common peptides (^**2**^). Differentiation between ACTC, ACTS and ACTA proteins was not possible on the basis of the data since the identified peptides were common to all of them (^**3**^). Differentiation between β-actin and γ-actin proteins was not possible on the basis of the data since the identified peptides were common to both isoforms (^**4**^).

We aimed at identifying proteins that interact with RACK1 interactions that occurr specifically in response to the activation of the cAMP signaling. To do so, we used the neuroblastoma SH-SY5Y cells as a cell model system and utilized “interactomics” approach in which RACK1 was immunoprecipitated (IPed) following activation of cAMP signaling by the adenylate cyclase activator, forskolin (FSK). Proteins that co-immunoprecipitated (co-IPed) with RACK1 were resolved by SDS-PAGE (Fig 1A) and submitted to mass spectrometry (MS) for identification. To rule out possible non-specific interactions, proteins recovered from the IgG control gel were also analyzed by MS. This strategy led to the identification of 5 different actin isoforms which we found to be enriched in the RACK1 IP lane as compared to the corresponding IgG control lane as shown by the higher number of identified peptides (Fig 1B). Among these actin isoforms, actin cytoplasmic 1 (β-actin) and actin cytoplasmic 2 (γ-actin) had the highest identification score than the 3 other actin isoforms (25.7 versus 11.2; Fig 1B). Due to the high protein sequence homology between β-actin and γ-actin isoforms (more than 98% identity due to only four amino acids substitutions within the N-terminus), only one peptide out of 13 allowed for discrimination between the β and γ isoforms. Together, these data raise the possibility that actin and particularly β/γ-actin binds to RACK1.

### RACK1 and β-actin are direct binding partners

**Fig 2:**
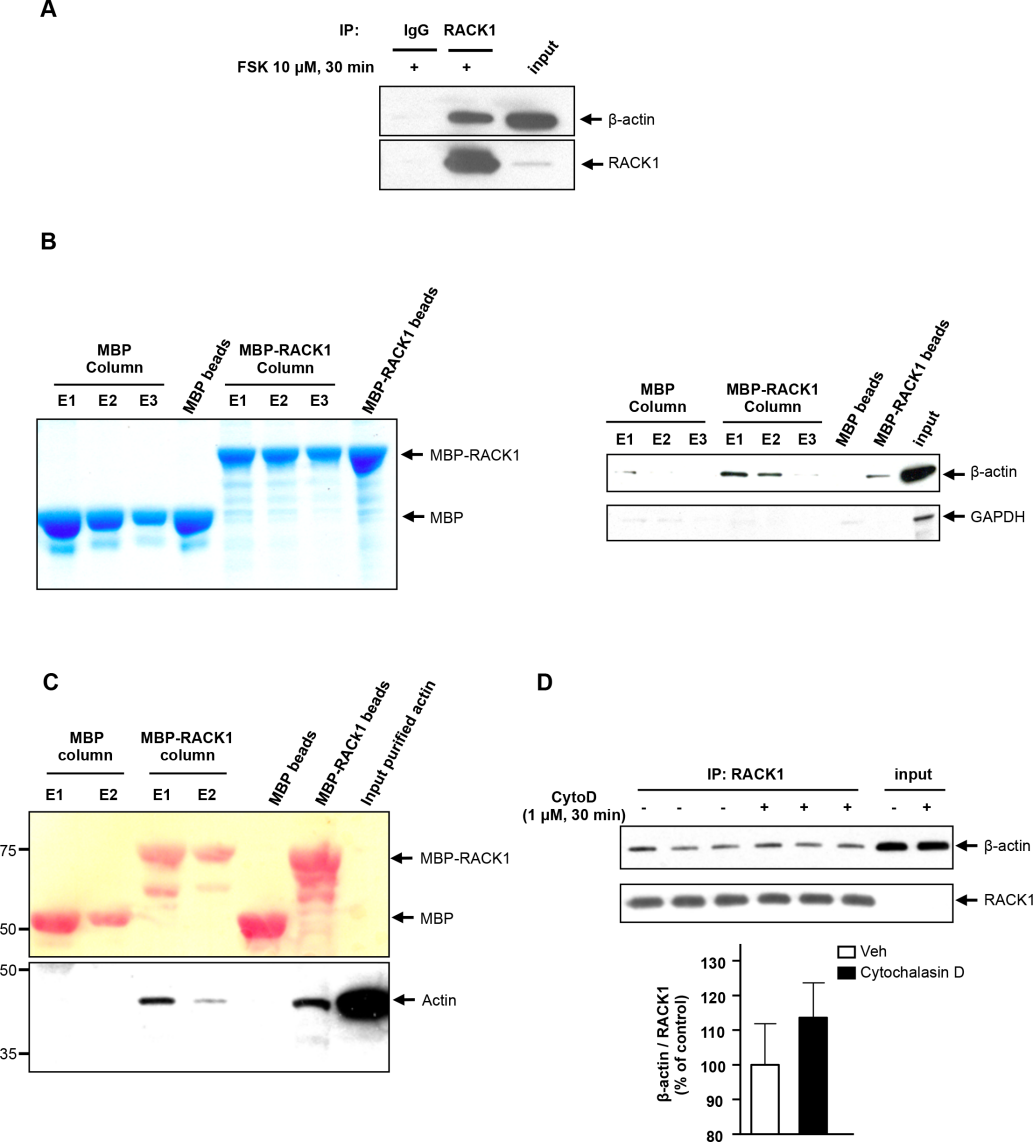
β-actin is a direct binding partner of RACK1. ***A*** SH-SY5Y cells were treated with 10 μM FSK for 30 min, and were then lysed in IP buffer. RACK1 was IPed from whole cell lysate and the co-IPed proteins were resolved by SDS-PAGE. Endogenous RACK1 and β-actin were analyzed by western blot analysis. ***B*** Recombinant MBP and MBP-RACK1 were immobilized on an amylose resin column and incubated with SH-SY5Y lysate previously treated with 10 μM FSK for 30 min. After washing, bound proteins were eluted 3 times with 50 mM maltose (E1, E2 and E3) or with loading buffer (MBP and MBP-RACK1 beads). Proteins were resolved by SDS-PAGE and the presence of β-actin and GAPDH was determined by western blot (right panel). The amount of MBP and MBP-RACK1 recovered after elution was also controlled with colloidal coomassie blue staining (left panel). n = 2. ***C*** The experiment was conducted as in panel ***B*** except that immobilized recombinant MBP and MBP-RACK1 were incubated with pure non-muscle actin. After washing, proteins were eluted twice with 50 mM maltose (E1 and E2) or with loading buffer (bead lanes). Eluted proteins were resolved by SDS-PAGE, transferred to nitrocellulose membrane and stained with Ponceau S to control the amount of MBP and MBP-RACK1 (upper panel). The presence of actin was subsequently determined by western blot analysis using a pan actin antibody (lower panel). ***D*** SH-SY5Y cells were incubated with vehicle or 1 μM cytochalasin D (cytoD) for 30 min, and then lysed in IP buffer. RACK1 was immunoprecipitated from whole cell lysate and proteins were resolved by SDS-PAGE. RACK1 and β-actin were revealed by western blot analysis. Histogram depicts the mean ratio of β-actin to RACK1, expressed as percent control ± SEM. Two-tailed unpaired t-test, p = 0.40. A n = 3, B and C n = 2, D n = 6.

We focused our studies on the β-actin isoform, and first confirmed that β-actin is indeed a RACK1 binding partner by using several complementary biochemical methods. First, SH-SY5Y cells were treated with FSK for 30 min, RACK1 was IPed from whole cell lysate, and the presence of β-actin in the RACK1 IP condition was examined by western blot analysis using an antibody that specifically detects β-actin. As shown in Fig 2A, we observed a specific interaction between endogenous RACK1 and β-actin in cells that were treated with FSK, validating the MS data presented in Fig 1. Next, we tested the ability of recombinant RACK1 to bind β-actin from FSK-activated cells. Specifically, recombinant RACK1 fused to maltose binding protein (MBP) or MBP was immobilized on an amylose resin column (Fig 2B, left panel). Equal amounts of cell lysates treated with FSK for 30 min were then applied to both columns, and the interaction of endogenous β-actin with the eluted MBP-RACK1 or MBP was tested by western blot analysis. We found that endogenous β-actin bound MBP-RACK1 but not MBP (Fig 2B, right upper panel). As an additional control, we also examined whether endogenous glyceraldehyde 3-phosphate dehydrogenase (GAPDH) was also eluted with the recombinant proteins and found that GAPDH did not interact with either MBP or MBP-RACK1 (Fig 2B right lower panel). Together, these data demonstrate that the association of RACK1 and β-actin is specific.

Next, we determined whether the interaction between RACK1 and β-actin was direct. To test this possibility, we carried out an *in vitro* binding assay in which the association between purified RACK1 and β-actin was examined. Recombinant MBP-RACK1 or MBP were immobilized on an amylose resin column and then incubated with purified non-muscle actin, which is composed of 85% and 15% β-actin and γ-actin respectively. Using pan-anti-actin antibodies, we observed that actin was specifically eluted with MBP-RACK1 but not MBP (Fig 2C). These data indicate that the association between RACK1 and β-actin is mediated through a direct protein-protein interaction.

To test whether RACK1 preferentially binds monomeric G-Actin or filamentous F-Actin, RACK1 was IPed from SH-SY5Y cells that were previously treated with 1 μM of cytochalasin D, a drug that induces the depolymerization of F-actin into G-actin [12]. We observed that treatment of cells with cytochalasin D did not alter the ability of β-actin to co-IP endogenous RACK1 (Fig 2D). These data indicate that in non-stimulated conditions, the association between RACK1/β-actin relies on the interaction of RACK1 with monomeric G-actin.

### Activation of the cAMP pathway increases the association between RACK1 and β-actin

**Fig 3:**
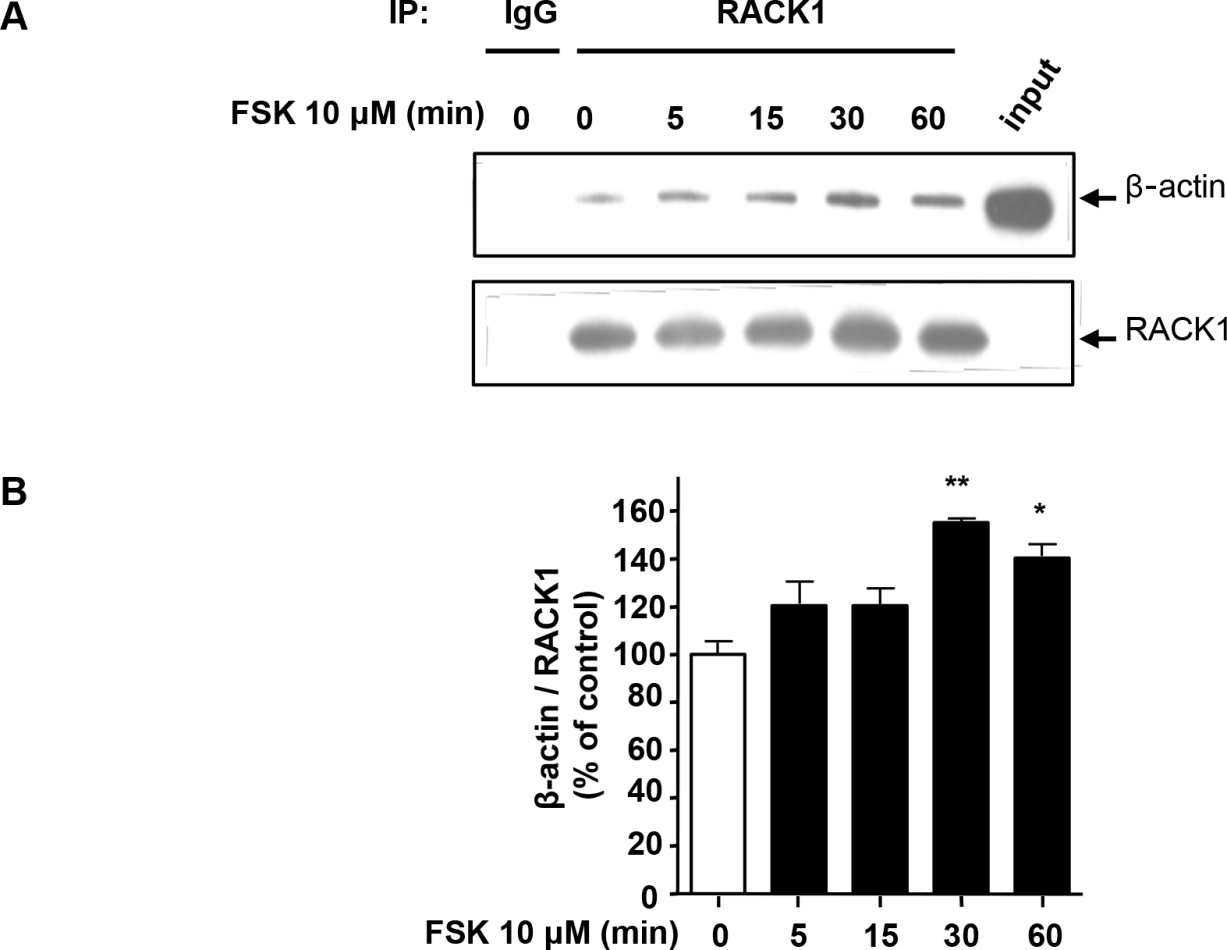
Activation of the cAMP pathway increases the association between RACK1 and β-actin. ***A*** SH-SY5Y cells were treated with vehicle or 10 μM FSK for the indicated duration, then lysed in IP buffer. RACK1 was IPed from whole cell lysate and proteins were resolved by SDS-PAGE. The presence of RACK1 and β-actin was determined by western blot analysis. ***B*** Quantification of ***A***. Histogram depicts the mean ratio of β-actin to RACK1, expressed as percent control (0 min) ± SEM. One-way ANOVA showed a significant effect of treatment [P = 0.002]. *p<0.05, **p<0.01 vs. 0 min, Newman-Keuls post-hoc analysis. n = 3.

Next, we examined whether activation of the cAMP pathway regulates the association between RACK1 and β-actin. To do so, endogenous RACK1 was IPed from SH-SY5Y cells treated with or without FSK, and the amount of β-actin co-IPed with RACK1 was measured by western blot analysis. As shown in Fig 3, we found that RACK1 and β-actin interacts both in the presence and absence of FSK stimulus. However, the activation of the cAMP pathway leads to a significant increase in β-actin interaction with RACK1 (Fig 3).

### Disruption of actin filament inhibits cAMP-mediated *BDNF* transcription

**Fig 4:**
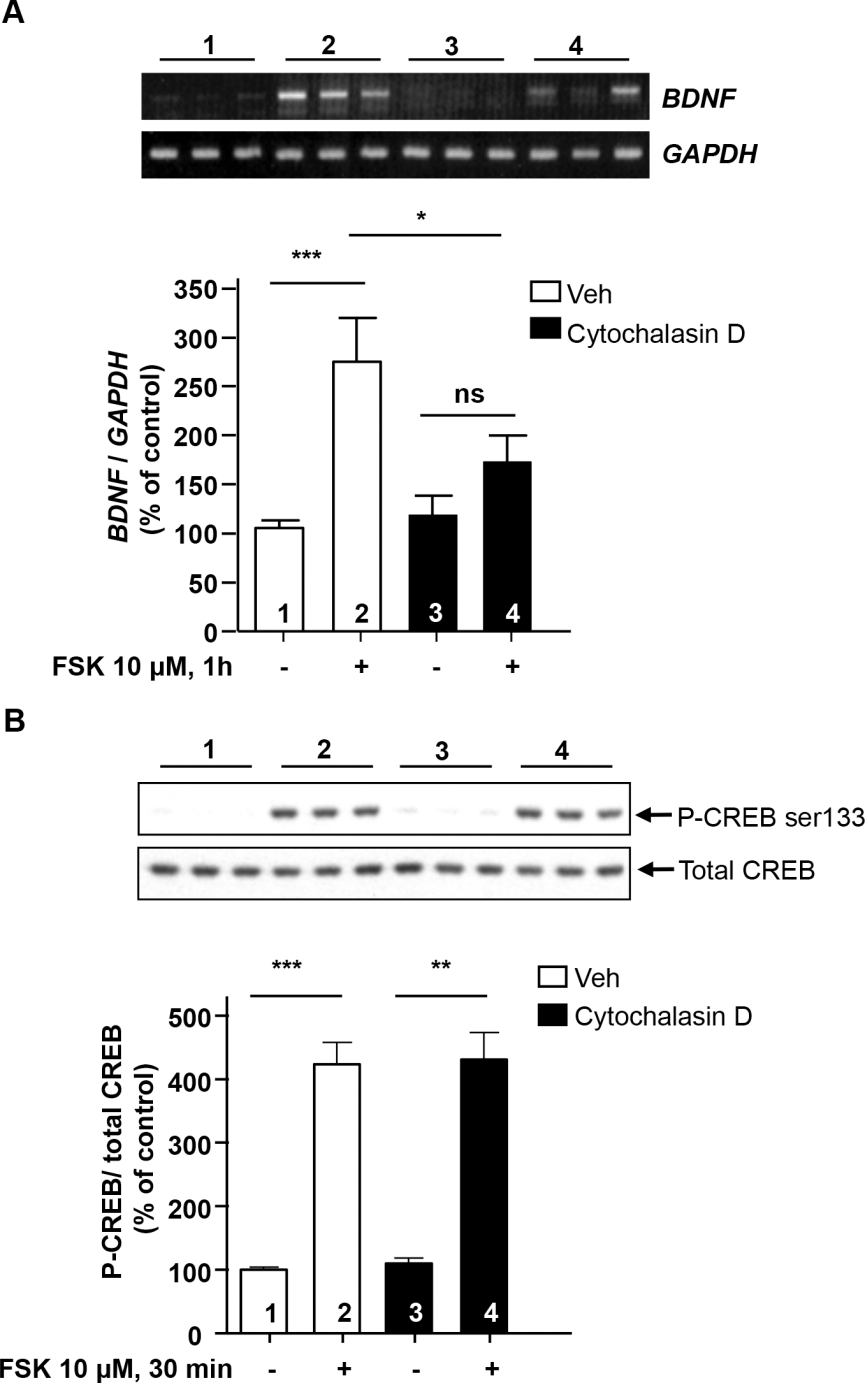
Disruption of actin filaments using cytochalasin D attenuates cAMP-mediated *BDNF* transcription without blocking the phosphorylation of CREB. ***A*** SH-SY5Y cells were incubated with vehicle or 1 μM cytochalasin D for 15 min prior to treatment with 10 μM FSK for 1 h. *BDNF* and *GAPDH* mRNA levels were analyzed by RT-PCR. Histogram depicts the mean ratio of *BDNF* to *GAPDH* expressed as percent control (vehicle) ± SEM. Two-way ANOVA shows an interaction between FSK and cytochalasin D [P = 0.049]. ***p<0.001, *p<0.05, ns p = 0.177, Newman-Keuls post-hoc analysis. ***B*** SH-SY5Y cells were incubated with vehicle or 1 μM cytochalasin D for 15 min prior to treatment with 10 μM FSK for 30 min, then lysed in IP buffer. Proteins were resolved by SDS-PAGE and the phosphorylation of CREB on serine 133 was examined by western blot analysis. Histogram depicts the mean ratio of phospho-CREB to total CREB, expressed as percent control (vehicle) ± SEM. Two-way ANOVA shows an effect of FSK [P < 0.001] but no effect of cytochalasin D [P = 0.756] and no interaction [P = 0.966]. Subsequent analysis by the method of contrasts (two-tailed unpaired t-test) detected a significant difference between control and FSK in both vehicle and cytochalasin D groups. **p = 0.002 and ***p<0.001. A n = 9, B n = 3.

Activation of the cAMP pathway results in the regulation of several cellular functions including modification of cytoskeletal dynamics in the cytosol [13] and activation of gene transcription in the nucleus [6–8, 14, 15], and RACK1 has been shown to play a central role in these processes [6, 9, 16]. We previously reported that activation of the cAMP pathway results in RACK1-dependent *BDNF* transcription in SH-SY5Y cells [6]. Therefore, we asked whether β-actin also plays a role in cAMP-mediated *BDNF* gene transcription. To address this question, we first examined whether alterations of actin function affect cAMP-mediated *BDNF* transcription. To do so, SH-SY5Y cells were treated with cytochalasin D, an inhibitor of actin polymerization, prior to activation of the cAMP pathway by FSK, and *BDNF* mRNA levels were measured by RT-PCR. As shown in Fig 4A, multiple comparisons analyses using Newman-Keul’s post hoc test show that following treatment of SH-SY5Y cells with cytochalasin D, activation of the cAMP cascade by FSK was unable to robustly promote *BDNF* transcription (see histogram in Fig 4A). Noteworthy, we obtained the same conclusion using Tukey, Holm-Sidak and Fisher’s LSD post hoc tests (data not shown). These data suggest that disruption of actin filaments using cytochalasin D attenuates cAMP-mediated induction of *BDNF* transcription.

To test whether cytochalasin D-mediated function was specific to *BDNF* transcription and was not due to a mere and global blockade of cAMP signaling, we examined phosphorylation level of the cAMP response element binding protein (CREB), a well-described substrate of protein kinase A [17], in response to FSK treatment with vehicle or cytochalasin D. We observed that disruption of actin filaments with cytochalasin D did not affect cAMP-mediated phosphorylation of CREB (Fig 4B). Taken together, these data raised the possibility that actin is involved in the mechanisms that promote cAMP-mediated *BDNF* transcription.

### RACK1 binds nuclear β-actin

**Fig 5:**
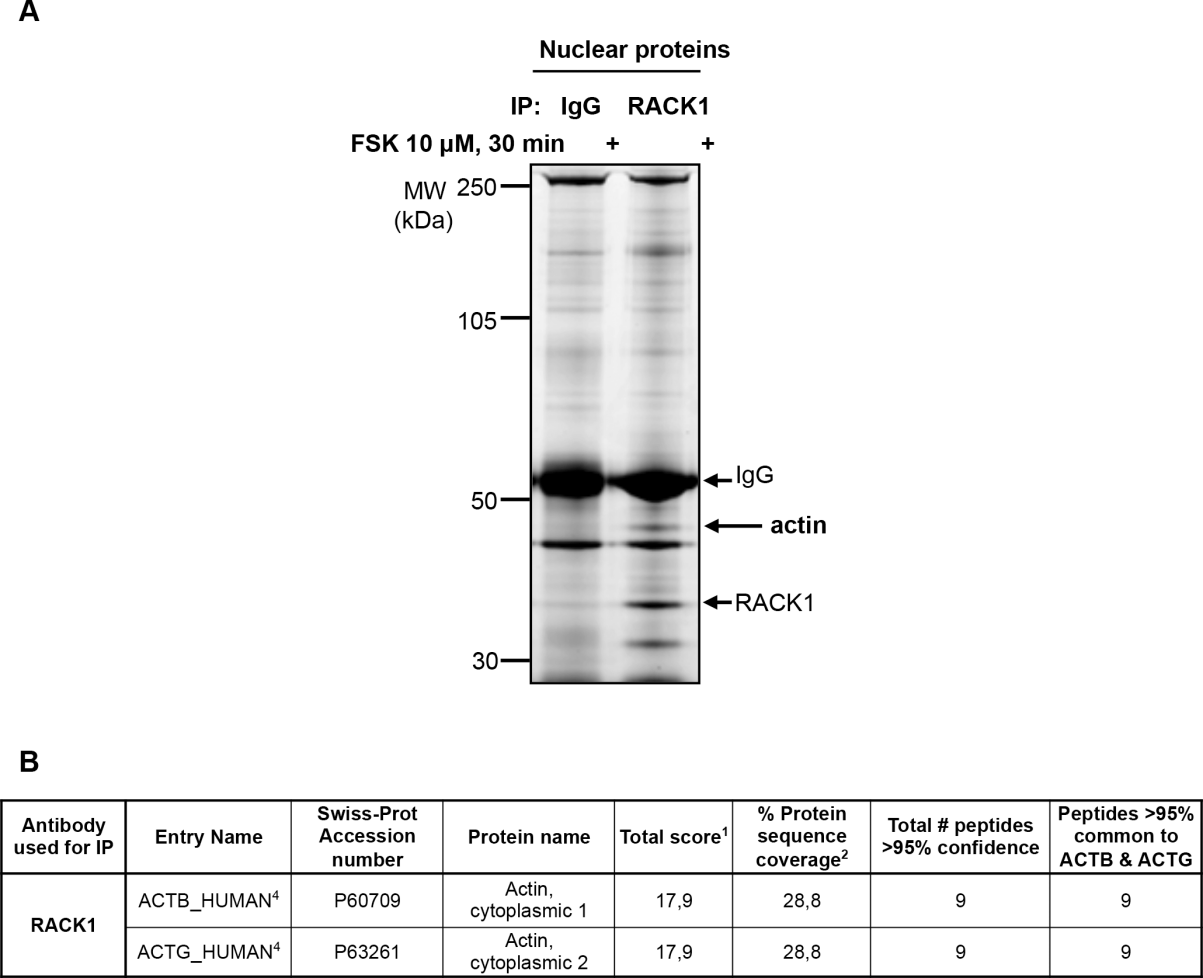
Nuclear association of RACK1 with β-actin. SH-SY5Y cells were treated with 10 μM FSK for 30 min, nuclear proteins were isolated and nuclear RACK1 was immunoprecipitated. Proteins were resolved by SDS-PAGE and the gel was stained with Deep Purple™ to visualize proteins. The gel slice indicated by an arrow was in-gel protein digested and resulting peptides were submitted to mass spectrometry (MS/MS) sequencing for protein identification. *B* Table shows the data leading to the identification of β-actin (ACTB) and γ-actin (ACTG) proteins. Numbers refer to the legend of Fig 1B.

Although actin is primarily known for its cytosolic function as a major component of the cytoskeleton, actin is also found in the nucleus [18, 19]. In this cell compartment, the monomeric β-actin isoform has been reported to regulate several stages of gene transcription including chromatin remodeling [18, 19]. In addition, β-actin has been shown to bind the promoters of both housekeeping and inducible genes [11, 20, 21]. We previously reported that, in response to cAMP pathway activation, RACK1 is present at the *BDNF* promoter IV, where it is required for chromatin remodeling and subsequent promoter activation [6]. Hence, we next hypothesized that nuclear β-actin contributes to cAMP-mediated *BDNF* transcription. To address this possibility, we first set out to examine whether RACK1 and β-actin interact in the nuclear compartment. To do so, SH-SY5Y cells were treated with FSK for 30 min, then nuclear proteins were isolated and nuclear RACK1 was IPed. Co-IPed proteins were resolved by SDS-PAGE and stained with Deep Purple^TM^. As shown in Fig 5A, we observed that a protein, whose molecular weight is similar to actin (around 42 kDa), was specifically recovered in RACK1 but not in the IgG control lanes. This protein was further identified as β/γ-actin by MS (Fig 5B), suggesting that RACK1 and actin also interact in the nuclear compartment.

### β-actin localizes at *BDNF* promoter IV following activation of the cAMP pathway in a mechanism that requires RACK1

**Fig 6:**
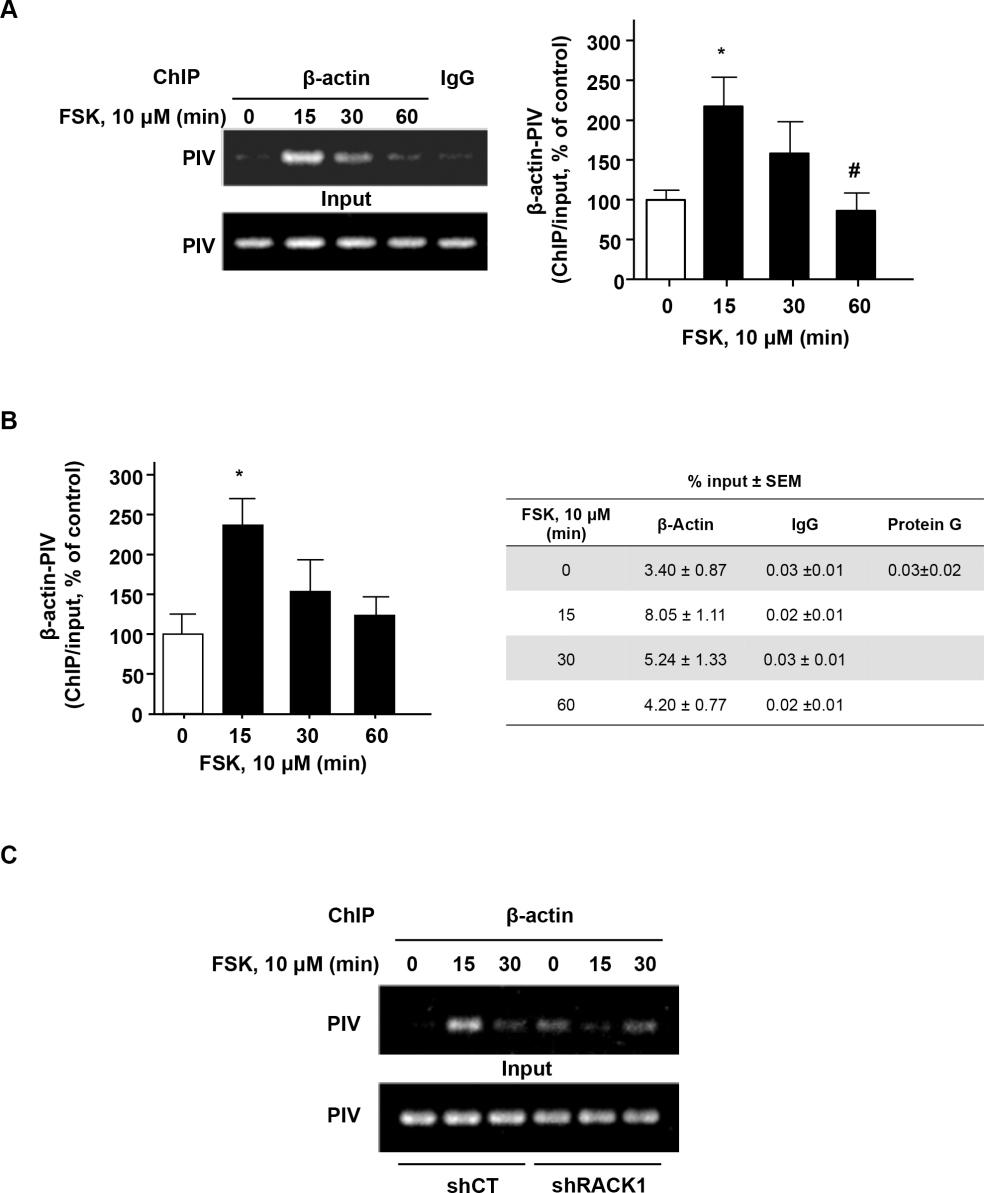
Activation of the cAMP pathway increases the association of β-actin with *BDNF* promoter IV via RACK1. SH-SY5Y cells were treated with vehicle or with 10 μm FSK for the indicated time points, then lysed for a ChIP assay with normal IgG or anti- β-actin antibody. The β-actin-associated *BDNF* promoter IV (PIV) in the ChIP samples was detected by semi quantitative PCR (***A***) and quantitative PCR (***B***). ***A*** The level of PIV in the input was analyzed in parallel. The histogram depicts the mean ratio of β-actin -associated PIV to input PIV expressed as percent control ± SEM. One-way ANOVA detected a significant effect of the treatment [P = 0.020]. *p<0.05 (0 min vs. 15 min) and #p<0.05 (15 min vs. 60 min), Newman-Keuls post-hoc analysis. *B* The table depicts the quantity of *BDNF* PIV in the β-actin, IgG control or Protein G ChIP samples, expressed as percent of input ± SEM. One-way ANOVA detected a significant effect of the treatment [P = 0.041]. *p<0.05 (0 min vs. 15 min), Newman-Keuls post-hoc analysis. *C* SH-SY5Y cells were infected with control adenovirus (shCT) or adenovirus expressing shRACK1 for 3 days before treatment with 10 μM FSK for the indicated time points. A ChIP assay was then conducted as described in ***A***. A n = 6, B n = 4 and C n = 2 per group.

As the activation of the cAMP pathway promotes RACK1 translocation to the nucleus where it associates with the *BDNF* promoter IV enabling subsequent promoter activation [6], we hypothesized that nuclear β-actin contributes to cAMP-mediated *BDNF* transcription by its association with RACK1 at the *BDN*F IV promoter. To address this possibility, we first examined whether β-actin was present at the promoter IV of *BDNF* upon activation of cAMP signaling. To do so, ChIP-PCR (Fig 6A) and ChIP-qPCR (Fig 6B) assays were performed to probe the presence of β-actin at *BDNF* promoter IV following activation of cAMP signaling by FSK. We observed that treatment of SH-SY5Y cells with FSK led to the binding of β-actin to *BDNF* promoter IV (Fig 6A and 6B).

Finally, to test whether RACK1 is required for the association of β-actin with *BDN*F IV promoter, the RACK1 gene was silenced using a previously validated shRNA sequence [6] and a β-actin ChIP assay was performed. We found that shRNA-mediated knockdown of RACK1 prevented the binding of β-actin to *BDNF* promoter IV in response to FSK treatment (Fig 6C). Together, these results suggest that cAMP-mediated binding of β-actin to *BDNF* promoter IV requires RACK1 and implicates β-actin in the mechanisms regulating cAMP-induced *BDNF* transcription.

## Discussion

In the present study, we report that β-actin is a new direct binding partner of the scaffolding protein RACK1, and provide evidence to suggest that this interaction is modulated by cAMP signaling. Our findings also indicate that the interaction between RACK1 and β-actin occurs in both the cytosolic and nuclear cell compartments. Finally, our data suggest that a functional interplay between β-actin and RACK1 is key for cAMP-induced *BDNF* transcription.

β-actin and γ-actin are the major actin isoforms in non-muscle cells [22]. Although several lines of evidence show that the two isoforms have distinct functions [22, 23], they are 98% identical and our MS data suggest that RACK1 interacts indistinctly with both isoforms, however, this possibility needs to be confirmed.

We found that recombinant RACK1 interacts with purified actin *in vitro* suggesting that the association between the two proteins is direct. In cells, non-muscle actin exists in two main functionally and structurally different forms: globular actin (G-actin) and filamentous (F-actin), which correspond to the monomeric and polymerized cytoskeletal actin, respectively [24, 25]. The polymerization of G-actin into F-actin is a fine-tuned process that requires implicates the presence of actin binding proteins or actin-related proteins (Arp) such as Arp2/3 or cofilin [26, 27]. The fact that we found that disruption of F-actin filaments by cytochalasin D did not affect RACK1/β-actin basal interaction indicates that in non-stimulated conditions, RACK1 is able to interact with G-actin.

We also show that RACK1/β-actin interaction is enhanced in response to adenylate cyclase activation. The main mode of transducing adenylate cyclase signal is via the activation of protein kinase A (PKA), thus, it is plausible that cAMP-mediated RACK1/β-actin interaction relies on a phosphorylation event, although RACK1 is not a direct substrate of PKA (Ron D., unpublished data). RACK1 subcellular location is sensitive to various stimuli [28, 29] including cAMP signaling [6, 8, 9], and thus, another possibility is that the activation of the cAMP pathway triggers the translocation of RACK1 towards actin microfilaments leading to the association of the two binding partners within the cytoskeletal structure. In a similar fashion, βIIPKC and RACK1 associate in response to stimuli prior to their translocation to a specific site [29].

The interaction between RACK1/β-actin raises the interesting possibility that RACK1 plays a role in the control of cytoskeleton dynamics. Interestingly, cAMP signaling can modulate cytoskeletal organization, for example by promoting migration and spreading of cells [13] and by contributing to neurite outgrowth in the nervous system [30]. Interestingly, RACK1 has been reported to play a role in the translation of β-actin mRNA in neurons [31], and several studies suggested an indirect involvement of RACK1 in the molecular mechanisms that participate in actin cytoskeletal dynamics [16, 32, 33], through its capacity to bind Src, PKC or focal adhesion kinase (FAK), key kinases whose activity controls the dynamics of microfilaments [34–36]. It is possible that, due to its scaffolding properties, RACK1 binding to the actin cytoskeleton brings proteins into close proximity that can then regulate cytoskeletal dynamics. Alternatively, cAMP-mediated binding of RACK1 to F-actin may competitively displace or prevent the binding of proteins such as Arp2/3 or cofilin that control the assembly and disassembly of actin filaments [27, 37]. In agreement with this hypothesis, we recently showed that RACK1 binds 14-3-3ζ [9], a protein that can regulate cytoskeleton dynamics by interacting with actin-filament-associated proteins such as cofilin [38]. Thus, our present work paves the way for the identification of additional roles for RACK1 in this cellular process.

In addition of being a major component of the cytoskeleton, polymeric actin is also present in the nucleus [39, 40] where it contributes to the dynamics of the nucleoskeleton [41, 42]. Our data raise the possibility that RACK1 interacts with nuclear β-actin. The possibility that RACK1 participates in actin nucleoskeleton dynamics is intriguing and merits further investigation. Similar to RACK1 [1], β-actin has reported to be a component of both the chromatin remodeling complex [43] and the transcriptional machinery [18]. We previously showed that nuclear RACK1 participates in cAMP-mediated exon IV *BDNF* expression [6]. In the present study, we show that like RACK1, β-actin binds promoter IV of *BDNF* with the same time course as RACK1 and in both cases the binding of the two proteins with the promoter IV of *BDNF* is cAMP-dependent ([6], and herein). Moreover, we observed that cAMP-dependent binding of β-actin to *BDNF* promoter IV is abolished when RACK1 gene is silenced. As RACK1 is required for cAMP-induced exon IV *BDNF* transcription [6], it is plausible that the association between RACK1 and β-actin enables the latter to bind to the *BDNF* promoter IV promoter, which then recruits histone acetyltransferase, permitting the initiation of transcription [18, 21]. The simultaneous binding of RACK1 and β-actin as a protein complex to *BDNF* promoter IV is an alternative possibility for the initiation of *BDNF* exon IV transcription. Interestingly, we also observed that the association of β-actin with *BDNF* promoter IV is transient suggesting that β-actin is released from the promoter following chromatin decondensation by acetylation of histone H4, which reaches a maximum 30 min after activation of cAMP signaling [6]. These potential mechanisms that enable RACK1 and β-actin to participate in exon IV BDNF transcription merit further investigation.

Human *BDNF* gene structure consists of nine functional promoters that separately control distinct *BDNF* exons which are differentially responsive to various stimuli [44]. We previously showed that although the cAMP pathway participates in the expression of *BDNF* exon I, IV and VI, RACK1 plays a specific role in the expression of *BDNF* exon IV only [6]. Therefore, an open question that requires further investigation is whether β-actin binds to other *BDNF* promoters and plays a role in the transcription of other *BDNF* exons.

Finally, the role of β-actin in gene transcription is not restricted to the stimulation of initiation. β-actin is also thought to be important for transcript elongation and transport [18, 19]. Therefore, the finding that RACK1 and β-actin interacts also opens new routes of investigation into a potential function of nuclear RACK1 in transcription elongation and the nucleo-cytoplasmic shuttling of mRNAs.

In summary, the present study reveals that the scaffolding protein RACK1 binds to β-actin in a cAMP-dependent manner and that a functional interplay between RACK1 and β-actin is key for the regulation of *BDNF* transcription. Therefore, our findings open a new route of investigation regarding the regulation of actin filaments and nuclear actin functions through RACK1 scaffolding properties in normal cell physiology and diseases.
